# Supplementary material for: Asymmetric Synthesis of Quaternary Hydantoins via a Palladium-Catalyzed Aza-Heck Cyclization
Source: J Am Chem Soc. 2025 Nov 14;147(49):44692–8. doi: 10.1021/jacs.5c16022 (PMC12703750; doi:10.1021/jacs.5c16022)
Supplement: Supplementary file 2 [file ja5c16022_si_002.zip › All NMR FID Files/S60/1/pdata/1/email_EM04-032-1_1_1.pdf]

PROTON8 CDCl3 /opt/nmrdata emeck 2

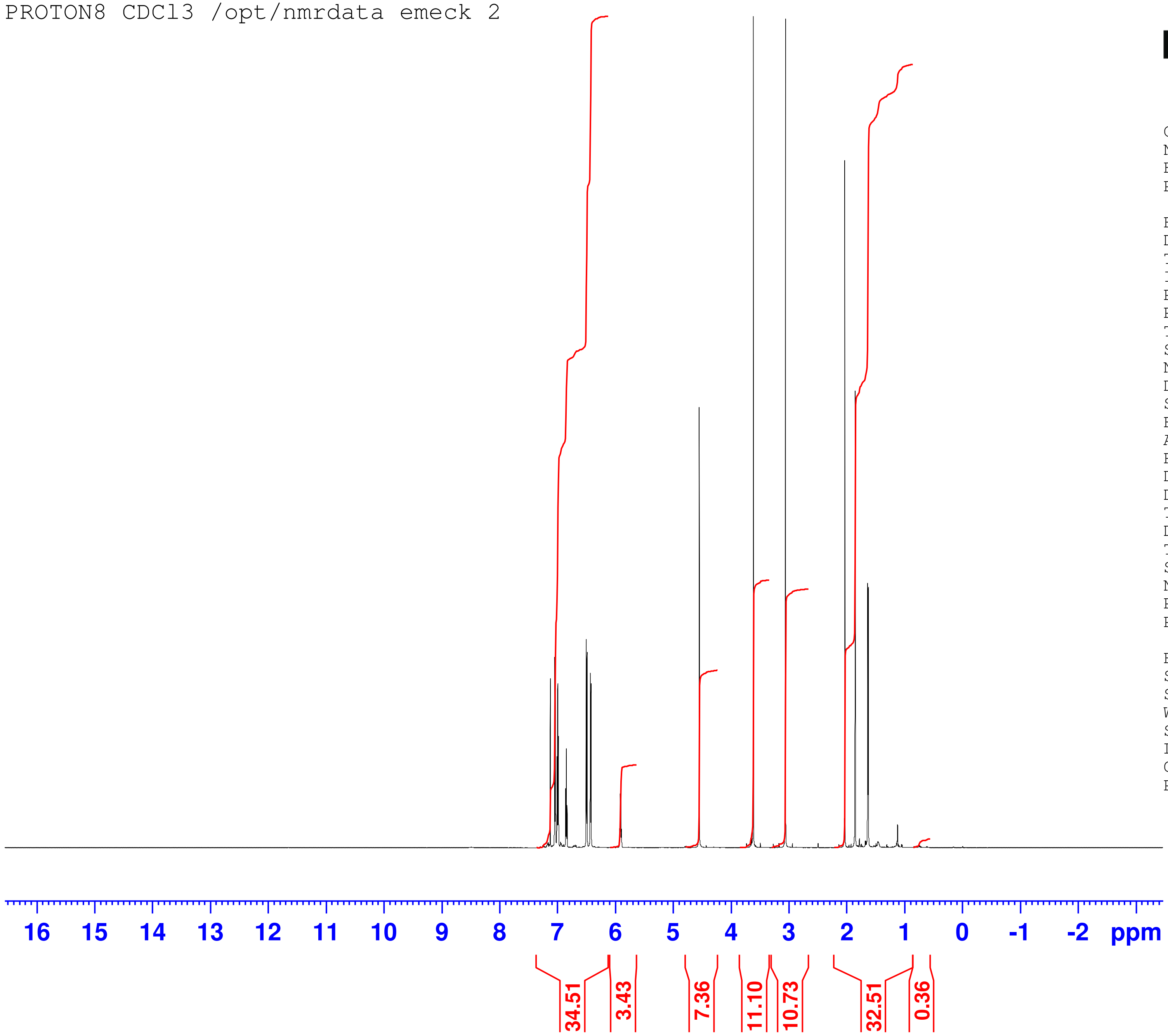

Current Data Parameters
NAME EM04-032-1
EXPNO 1
PROCNO 1

F2 - Acquisition Parameters
Date_ 20230320
Time 15.04 h
INSTRUM spect
PROBHD Z114607_0069 (
PULPROG zg30
TD 65536
SOLVENT CDCl3
NS 16
DS 2
SWH 12019.230 Hz
FIDRES 0.366798 Hz
AQ 2.7262976 sec
RG 90.5
DW 41.600 usec
DE 11.53 usec
TE 300.0 K
D1 1.00000000 sec
TD0 1
SFO1 600.3240221 MHz
NUC1 1H
P1 11.15 usec
PLW1 26.00000000 W

F2 - Processing parameters
SI 65536
SF 600.3200918 MHz
WDW EM
SSB 0
LB 0.30 Hz
GB 0
PC 1.00
